# Identification of Infertility-Associated Topologically Important Genes Using Weighted Co-expression Network Analysis

**DOI:** 10.3389/fgene.2021.580190

**Published:** 2021-02-03

**Authors:** Jingni Wu, Xiaomeng Xia, Ye Hu, Xiaoling Fang, Sandra Orsulic

**Affiliations:** ^1^Department of Obstetrics and Gynecology, The Second Xiangya Hospital, Central South University, Changsha, China; ^2^Department of Obstetrics and Gynecology, David Geffen School of Medicine, University of California, Los Angeles, Los Angeles, CA, United States

**Keywords:** infertility in endometriosis, hub lncRNA, hub mRNA, WGCNA, biomarker

## Abstract

Endometriosis has been associated with a high risk of infertility. However, the underlying molecular mechanism of infertility in endometriosis remains poorly understood. In our study, we aimed to discover topologically important genes related to infertility in endometriosis, based on the structure network mining. We used microarray data from the Gene Expression Omnibus (GEO) database to construct a weighted gene co-expression network for fertile and infertile women with endometriosis and to identify gene modules highly correlated with clinical features of infertility in endometriosis. Additionally, the protein–protein interaction network analysis was used to identify the potential 20 hub messenger RNAs (mRNAs) while the network topological analysis was used to identify nine candidate long non-coding RNAs (lncRNAs). Functional annotations of clinically significant modules and lncRNAs revealed that hub genes might be involved in infertility in endometriosis by regulating G protein-coupled receptor signaling (GPCR) activity. Gene Set Enrichment Analysis showed that the phospholipase C-activating GPCR signaling pathway is correlated with infertility in patients with endometriosis. Taken together, our analysis has identified 29 hub genes which might lead to infertility in endometriosis through the regulation of the GPCR network.

## Introduction

Endometriosis, a gynecological disorder characterized by the growth of endometrial glands and stroma outside the uterus, is clinically highly associated with infertility. However, the mechanisms of infertility in endometriosis remain unclear. Increasing evidence has suggested that endometriosis patients have an abnormal endometrial environment, such as dysregulated hormone levels and activated inflammatory factors, which is unfavorable for embryo implantation and pregnancy progression (de Ziegler et al., 2010; Lessey and Kim, 2017). However, some women with endometriosis can conceive without difficulty while others are infertile. Hence, infertility in endometriosis appears to be a complex multifactorial clinical condition. Current medical treatments for endometriosis are not effective against infertility, and surgical treatment may induce the failure of ovarian function. Identifying and understanding the molecular mechanisms of infertility in endometriosis will facilitate the development of early diagnostic criteria and therapeutic targets for infertility in women with endometriosis.

Microarray and high-throughput sequencing technologies combined with the development of bioinformatic algorithms have aided in the discovery of many potential molecular biomarkers for various diseases and conditions. Previously, most studies primarily focused on gene expression differences between different sample groups, ignoring the intrinsic relationship between genes. Networks, such as the co-expression network and protein–protein interaction (PPI) network, can provide a straightforward representation of gene interactions. Additionally, application of structure network algorithms can identify function-specific modules or sub-structures in these biological networks based on topological importance, such as correlation with clinical traits, degree, edge-clustering coefficient, or K-core level (Chen et al., 2014; Bu et al., 2021). However, to avoid the possible bias and limitations associated with single-network analysis, integrative analysis of multiple independent networks is recommended (Chen et al., 2014; Nangraj et al., 2020). The utilization of multiple networks has been shown to improve the understanding of the full spectrum of interactions, prioritize biomarkers for targeted therapy, and identify complex biological activities (Guney et al., 2016; Mahapatra et al., 2018). Weighted gene co-expression network analysis (WGCNA) is used to cluster highly correlated genes into the same co-expression module in order to further investigate the relationship between the module and disease types/clinical phenotypes. Therefore, WGCNA is able to identify biologically relevant modules, potential diagnostic biomarkers, and therapeutic targets. Recent examples of effective applications of WGCNA include the identification of hub genes associated with lung squamous cell carcinoma as well as hub genes of the perineural invasion phenotype in head and neck squamous cell carcinoma (Zhang et al., 2019; Gao et al., 2020). The PPI network reveals physical binding between protein pairs and uncovers the molecular mechanisms behind these interactions by following a pattern of small-world network from the shortest path, proximity to the center, and average aggregation coefficient (Zheng et al., 2019). Molecular Complex Detection (MCODE) has been used to illuminate the most critical genes and finest clusters in the PPI network based on the k-core algorithm. Li et al. (2015) successfully used the integration of the gene co-expression network with the PPI network for mining candidate hub genes for Alzheimer’s disease, and Feng et al. (2019) performed pathway analysis for microRNAs in type 2 diabetes mellitus by integrating gene co-expression data and PPI network information. Our goal was to apply WGCNA and integrated networks to identify functionally relevant molecular pathways associated with infertility in endometriosis.

Long non-coding RNAs (lncRNAs) are non-coding transcript clusters longer than 200 nucleotides. Recently, lncRNAs have been shown to play important roles in various cellular functions, including epigenetic regulation, transcription, and cell cycle control, and are emerging as potential diagnostic and therapeutic biomarkers for diseases (Fatica and Bozzoni, 2014; Yin et al., 2020). Previous studies have revealed that, compared to normal controls, eutopic endometria from infertile women with endometriosis present aberrant molecular expression, such as decreased expression of lncRNA H19, which might regulate the H19/Let-7/IGF1R pathway contributing to impaired endometrium receptivity for pregnancy in women with endometriosis (Ghazal et al., 2015). However, the expression pattern and roles of lncRNAs in infertility in women with endometriosis remain unknown.

**FIGURE 1:**
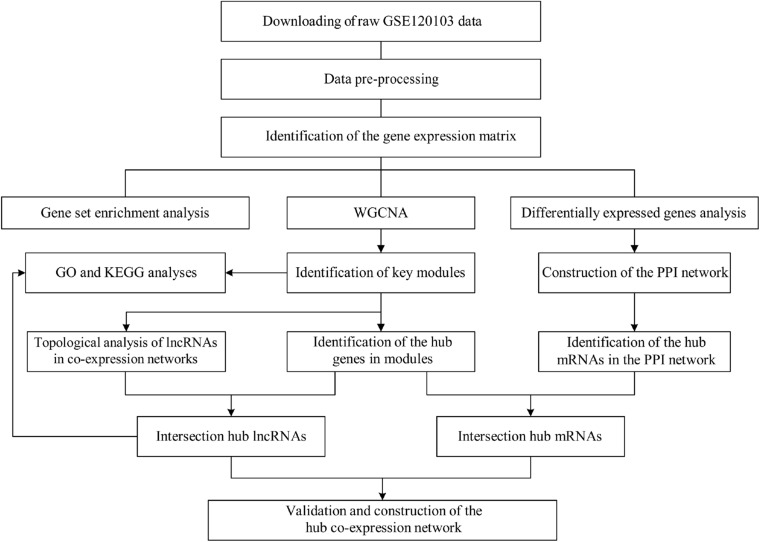
Flowchart of strategy used in this study for data preparation, pre-processing, and analysis. WGCNA, weighted gene co-expression analysis; GO, Gene Ontology; KEGG, Kyoto Encyclopedia of Genes and Genomes; PPI, protein–protein interaction; lncRNA, long non-coding RNA; mRNA, messenger RNA.

In this study, we evaluated potential biomarkers in the diagnosis and treatment of infertility in endometriosis patients by comparing differential expression profiles of lncRNAs and mRNAs in fertile and infertile patients with endometriosis. Then, we used bioinformatics algorithms, including WGCNA, PPI, and topological analyses, to identify hub lncRNA and mRNAs and their functions. After removing genes that were also differentially expressed between fertile and infertile patients without endometriosis, we identified hub genes specific to endometriosis-associated infertility (EAI). Our study might provide new insights into the molecular mechanisms of infertility in endometriosis. The flowchart of the analyses is shown in Figure 1.

## Materials and Methods

### Data Collection and Pre-processing

The microarray dataset GSE120103 analyzing the gene expression profile of infertility in endometriosis (Bhat et al., 2019) was downloaded from the Gene Expression Omnibus (GEO) database. Microarray analysis in the GSE120103 dataset was performed on the GPL6480 platform (Agilent-014850 Whole Human Genome Microarray 4 × 44K G4112F, Santa Clara, CA, United States). The dataset consisted of 36 samples that included nine fertile women without ovarian endometriosis (OE), nine infertile women without OE, nine fertile women with stage IV OE, and nine infertile women with stage IV OE. Both fertile and infertile patients with stage IV OE had no evidence of recurrence or any other endocrinological disorder or other complications of comorbidities. All patients were in the secretory menstrual phase and enrolled for surgical intervention. To recognize hub genes and co-expression networks associated with infertility in endometriosis patients, we selected 18 samples (nine fertile and nine infertile) with stage IV OE. Hub genes that were also differentially expressed in fertile and infertile women without endometriosis were screened out to explore the EAI-associated genes. All data were pre-processed by linear models for the microarray data (limma) package (Ritchie et al., 2007), including background correct function and avereps function, to correct for the background and summarize the probes.

### Differentially Expressed Genes Screening

The R package limma was applied to identify differentially expressed genes (DEGs), including differentially expressed mRNAs (DEMs) and differentially expressed lncRNAs (DELs), in different comparison groups. A false discovery rate (FDR) < 0.05 and | log_2_FC| ≥ 1 were defined as the cut-off criteria for screening DEGs.

### Gene Set Enrichment Analysis

Gene Set Enrichment Analysis (GSEA) is a computational method used to annotate gene functions. It evaluates whether a previously defined gene set is statistically significant between two biological states. The “ClusterProfiler” package in R^1^ was used to perform GSEA based on the Gene Ontology (GO) and Kyoto Encyclopedia of Genes and Genomes (KEGG) databases for all expressed genes. The threshold for screening differentially expressed gene sets was set as FDR < 0.05.

### Weighted Gene Co-expression Network Construction

The WGCNA package in R was used to establish the scale-free co-expression network (Langfelder and Horvath, 2008) for all expressed lncRNAs and mRNAs from fertile and infertile women with endometriosis. To ensure the network was reliable, unqualified genes were removed. An appropriate soft threshold power (β) was selected based on a scale-free topology criterion with which adjacencies between all genes in the module were calculated by a power function. Then, the adjacency matrix was transformed into the topological overlap matrix (TOM). TOM measures connectivity of paired genes to all other network-generated genes. Higher TOM values show that paired genes may be highly correlated with each other and connect with many shared genes (Li and Horvath, 2007; Yip and Horvath, 2007). This correlation method is more powerful than the Spearman/Pearson correlation, thus creating more robust co-expression relationships (Li and Horvath, 2007; Mähler et al., 2017). The genes were clustered hierarchically according to the TOM-based dissimilarity (1-TOM) measurement. The highly connected genes were then grouped into the same module.

### Clinically Significant Modules Identification

After the clinical information was imported into the network, its correlation with modules was investigated by the WGCNA module–trait relationship analysis. The modules most relevant to the clinical phenotypes could be identified. Here, we were interested in the infertility-associated yellow and blue modules.

### Functional Annotations of the Significant Modules

To explore the functional annotations of the genes in the yellow and blue modules, we used Metascape^2^ can integrate several data resources, including GO, KEGG, and UniProt, to annotate gene function (Zhou et al., 2019). Terms with *P* < 0.01, count ≥3, and enrichment factor >1.5 were considered statistically significant (Li et al., 2019).

### Construction of the PPI Network and Identification of Hub mRNAs

After identifying the clinically significant modules, we calculated the gene significance (GS) and module membership (MM) of each gene in the modules. The module eigengene was the most important component of the module’s gene expression matrix. GS was defined as the correlation between the gene and clinical phenotype of interest. MM represents the association of gene expression profile with module eigengene. We set the threshold of | MM| > 0.8 and | GS| > 0.8 for screening candidate hub genes (mRNAs and lncRNAs) that strongly associated with infertility in a module (Dai et al., 2020). A search tool database for the retrieval of interacting genes (STRING)^3^ was used to construct the PPI network based on the most significantly regulated DEMs. The PPI network was visualized by Cytoscape and further screened by MCODE (cut-off MCODE score = 10) (Liu et al., 2019) to identify candidate hub DEMs. Finally, we used Venn diagrams to identify the common hub mRNAs screened from the PPI network and module. The common hub mRNAs were defined as the hub mRNAs.

### Topological Analysis of the Co-expression Network and Selection of Hub lncRNAs

With the threshold of TOM-based | correlation coefficient| > 0.4, the weighted gene co-expression networks of the yellow and blue modules were visualized by Cytoscape. The topological analysis of lncRNAs was conducted to explore the central nodes of these networks. Generally, a higher degree indicates that the node is involved in more interactions. A higher betweenness suggests that the node acts as a bridge connecting different network modules. A better closeness indicates that the node is likely to be the center of the network (Özgür et al., 2008). Cytoscape with CentiScaPe 2.2 plug-in was applied to calculate the topological parameters (degree, betweenness, and closeness) in our analysis. The top-ranked lncRNAs (top five were shown in our study) in degree, betweenness, and closeness of the co-expression network and the previously analyzed hub lncRNAs from each module were compared. The common hub lncRNAs in the topological analysis and module were identified as the hub lncRNAs.

### Validation of Hub Genes

The GSE26787 dataset was used for validation of the hub mRNAs. The GSE26787 dataset includes 10 infertile women with recurrent miscarriages or implantation failures and five fertile control patients. We used the “ggplot2” (Ito and Murphy, 2013) R package to show the expression of the identified infertility-associated hub genes.

### Functional Prediction of lncRNAs

Long non-coding RNAs may regulate the expression of neighboring and distant protein-coding genes (Bonasio and Shiekhattar, 2014). To clarify the biological roles of the hub lncRNAs, the lncRNA co-expressed mRNAs calculated by WGCNA were analyzed by the plug-in ClueGO in Cytoscape for GO biological processes and KEGG pathways. ClueGO was used to visualize the relationship between the genes and GO terms. A *P* value < 0.05 was set as the screening condition.

## Results

### Identification of DEGs and Gene Set Enrichment Analysis

**FIGURE 2:**
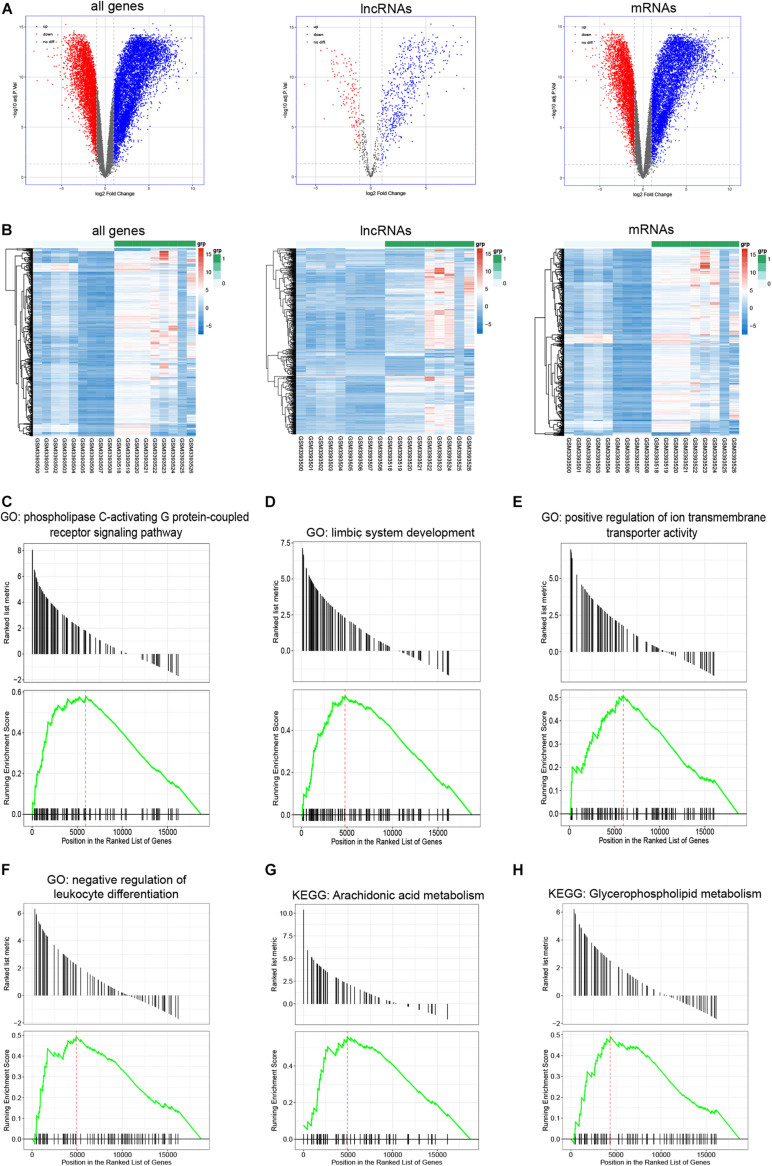
Identification of DEGs and Gene Set Enrichment Analysis. **(A,B)** Volcano plots of differentially expressed genes that include lncRNAs and mRNAs (DEGs), differentially expressed lncRNAs (DELs), and differentially expressed mRNAs (DEMs) as well as heatmaps of the top 500 genes (including lncRNAs and mRNAs), top 500 lncRNAs, and top 500 mRNAs based on the value of |logFC| in infertile and fertile women with endometriosis. **(C–H)** Gene Set Enrichment Analysis (GSEA)-identified biological processes with significant enrichment in infertile patients with endometriosis.

A total of 12,282 DEGs (7,671 upregulated and 4,611 downregulated), including 665 lncRNAs (529 upregulated and 136 downregulated), and 11,617 mRNAs (7,142 upregulated and 4,475 downregulated), were identified between infertile and fertile women with endometriosis. The volcano plot and heatmap show the variation of DEGs (Figures 2A,B).

To annotate gene functions, we performed GSEA for all expressed genes; 1,455 significantly enriched gene sets were obtained. Figures 2C–H show the most enriched ontology biological processes and enriched pathways, which include the phospholipase C-activating G protein-coupled receptor signaling pathway, positive regulation of ion transmembrane transporter activity, and negative regulation of the leukocyte differentiation pathway.

### Weighted Gene Co-expression Network Construction

**FIGURE 3:**
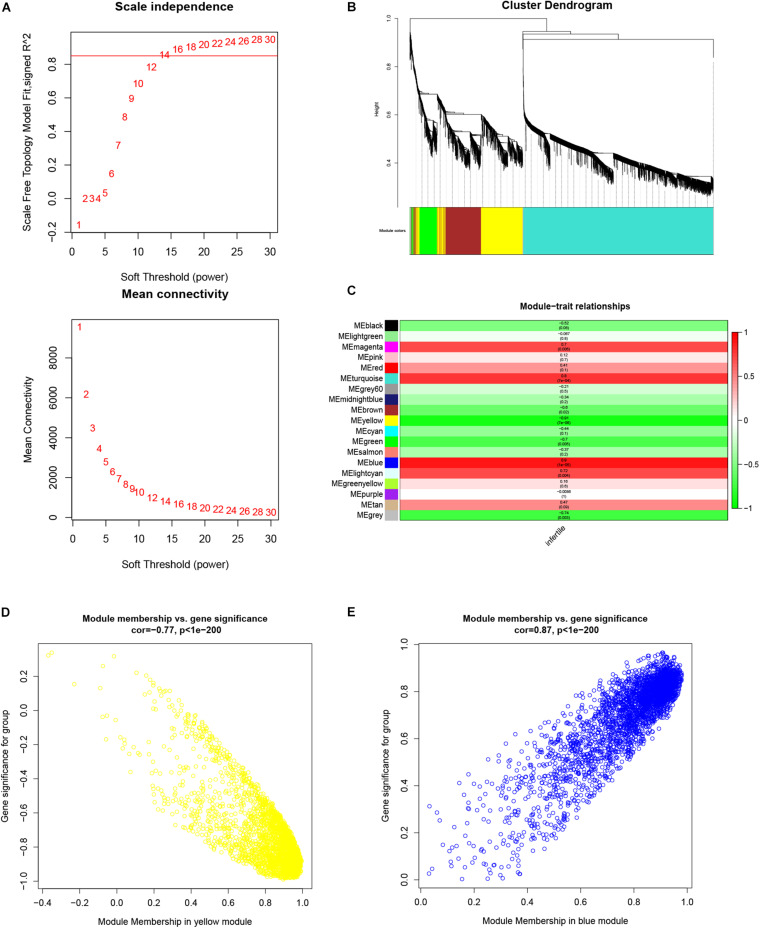
Weighted gene co-expression network analysis of lncRNAs and mRNAs associated with infertility in women with endometriosis. **(A)** Analysis of the scale-free fit index and the mean connectivity for various soft threshold powers (β). **(B)** Dendrogram for all expressed lncRNAs and mRNAs clusters based on a dissimilarity measure (1-TOM). **(C)** Determination of module–trait relationship of infertility in endometriosis. Each row indicates a module eigengene (the principal component of gene expression), while the heatmap represents a clinical trait of infertility in endometriosis. **(D,E)** Scatterplots of module eigengenes related to infertility in women with endometriosis in the yellow and blue co-expression modules.

To identify all co-expressed genes, we chose β = 12 (scale-free *R*^2^ = 0.85) as the soft threshold to construct a scale-free weighted gene co-expression network (Figure 3A). lncRNAs and mRNAs with similar expression patterns were assigned to co-expression modules; 19 co-expression modules were identified and are shown in different colors (Figure 3B).

### Identification of Clinically Significant Modules and Functional Annotations

The module-trait relationship is shown in Figure 3C. Of the 19 modules, the yellow module was the most negatively correlated with infertility in women with endometriosis (*R* = −0.91, *p* = 7 × 10^–6^), while the blue module was the most positively correlated with infertility in women with endometriosis (*R* = 0.9, *p* = 1 × 10^–5^). Therefore, we chose the yellow and blue modules as the clinically relevant modules. Subsequent GO analysis revealed that the most enriched biological process in the yellow module was mRNA processing (Supplementary Figure 1A), while the biological process enriched in the blue module was meiotic cell cycle nuclear division (Supplementary Figure 1C). KEGG analysis revealed that the most enriched pathways in the yellow module were the autophagy and estrogen signaling pathway (Supplementary Figure 1B), while the most enriched pathways in the blue module were cell cycle and DNA replication (Supplementary Figure 1D). These functional annotations for the yellow and blue modules are listed in Tables 1, 2, respectively. Our findings indicate that genes in the yellow and blue modules may play crucial roles in the pathogenesis of infertility in women with endometriosis.

**TABLE 1:** The GO and KEGG pathway analysis of genes in the yellow module.

Category	Term	Description	Log (*p*-value)
GO biological processes	GO:0006397	mRNA processing	−13.1387329
GO biological processes	GO:0051603	Proteolysis involved in cellular protein catabolic process	−11.941189
GO biological processes	GO:0022411	Cellular component disassembly	−9.70675281
GO biological processes	GO:0016570	Histone modification	−9.19579349
GO biological processes	GO:0006914	Autophagy	−8.37033415
GO biological processes	GO:0042176	Regulation of protein catabolic process	−7.74011458
GO biological processes	GO:0007346	Regulation of mitotic cell cycle	−7.19546552
GO biological processes	GO:0070646	Protein modification by small protein removal	−6.99274833
GO biological processes	GO:1903827	Regulation of cellular protein localization	−6.91574071
GO biological processes	GO:0016055	Wnt signaling pathway	−6.42998855
GO biological processes	GO:0045022	Early endosome to late endosome transport	−6.09245453
GO biological processes	GO:0019439	Aromatic compound catabolic process	−6.06027019
GO biological processes	GO:0034660	ncRNA metabolic process	−5.94825625
GO biological processes	GO:0034976	Response to endoplasmic reticulum stress	−5.86412414
GO biological processes	GO:1903008	Organelle disassembly	−5.82566573
GO biological processes	GO:0022613	Ribonucleoprotein complex biogenesis	−5.76102653
GO biological processes	GO:0006281	DNA repair	−5.74297374
GO biological processes	GO:1990778	Protein localization to cell periphery	−5.5607251
GO biological processes	GO:0043254	Regulation of protein complex assembly	−5.43340442
GO biological processes	GO:0080135	Regulation of cellular response to stress	−5.34445314
KEGG pathway	hsa03040	Spliceosome	−8.58414093
KEGG pathway	hsa04140	Autophagy–animal	−5.26931505
KEGG pathway	hsa04142	Lysosome	−3.3383058
KEGG pathway	hsa04962	Vasopressin-regulated water reabsorption	−3.27003669
KEGG pathway	hsa03015	mRNA surveillance pathway	−3.24114684
KEGG pathway	hsa03050	Proteasome	−3.1516681
KEGG pathway	hsa00511	Other glycan degradation	−3.05682556
KEGG pathway	hsa03420	Nucleotide excision repair	−2.86058099
KEGG pathway	hsa04915	Estrogen signaling pathway	−2.79128101
KEGG pathway	hsa04144	Endocytosis	−2.75350872
KEGG pathway	hsa04621	NOD-like receptor signaling pathway	−2.63019526
KEGG pathway	hsa00561	Glycerolipid metabolism	−2.60170601
KEGG pathway	hsa00230	Purine metabolism	−2.59658367
KEGG pathway	hsa04141	Protein processing in endoplasmic reticulum	−2.52148975
KEGG pathway	hsa04070	Phosphatidylinositol signaling system	−2.47102038
KEGG pathway	hsa04120	Ubiquitin-mediated proteolysis	−2.43043309
KEGG pathway	hsa04371	Apelin signaling pathway	−2.43043309
KEGG pathway	hsa04210	Apoptosis	−2.36203062
KEGG pathway	hsa05169	Epstein-Barr virus infection	−2.25136927
KEGG pathway	hsa04150	mTOR signaling pathway	−2.24370941

*GO biological processes, Gene Ontology analysis of biological process; KEGG, Kyoto Encyclopedia of Genes and Genomes.*

**TABLE 2:** The GO and KEGG pathway analysis of genes in the blue module.

Category	Term	Description	Log (*p*-value)
GO biological processes	GO:0051321	Meiotic cell cycle	−11.2637016
GO biological processes	GO:0000280	Nuclear division	−9.21239795
GO biological processes	GO:0044786	Cell cycle DNA replication	−8.07277536
GO biological processes	GO:0051301	Cell division	−7.722935
GO biological processes	GO:0022412	Cellular process involved in reproduction in multicellular organism	−7.20796891
GO molecular functions	GO:0015077	Monovalent inorganic cation transmembrane transporter activity	−6.4096854
GO biological processes	GO:0006268	DNA unwinding involved in DNA replication	−6.00120718
GO molecular functions	GO:0015291	Secondary active transmembrane transporter activity	−5.94025842
GO biological processes	GO:0009566	Fertilization	−5.79872588
GO biological processes	GO:0048664	Neuron fate determination	−5.60456786
GO biological processes	GO:0071103	DNA conformation change	−5.52974904
GO biological processes	GO:0050953	Sensory perception of light stimulus	−4.95474684
GO biological processes	GO:0006820	Anion transport	−4.3658481
GO biological processes	GO:0006281	DNA repair	−4.33202885
GO molecular functions	GO:0004222	Metalloendopeptidase activity	−4.32958972
GO biological processes	GO:0048839	Inner ear development	−4.21246032
GO molecular functions	GO:0005104	Fibroblast growth factor receptor binding	−4.20391833
GO biological processes	GO:0019730	Antimicrobial humoral response	−4.01810702
GO molecular functions	GO:0003774	Motor activity	−3.93572584
GO biological processes	GO:0009314	Response to radiation	−3.75992358
KEGG pathway	hsa04110	Cell cycle	−8.16036184
KEGG pathway	hsa04976	Bile secretion	−5.32999244
KEGG pathway	hsa03030	DNA replication	−4.46482567
KEGG pathway	hsa04080	Neuroactive ligand-receptor interaction	−4.27513306
KEGG pathway	hsa05034	Alcoholism	−3.18218307
KEGG pathway	hsa03460	Fanconi anemia pathway	−2.89472976
KEGG pathway	hsa04975	Fat digestion and absorption	−2.62975136
KEGG pathway	hsa05218	Melanoma	−2.58189181
KEGG pathway	hsa05203	Viral carcinogenesis	−2.55804795
KEGG pathway	hsa05033	Nicotine addiction	−2.22606437
KEGG pathway	hsa04060	Cytokine–cytokine receptor interaction	−2.07603943
KEGG pathway	hsa04742	Taste transduction	−2.07554483
KEGG pathway	hsa03410	Base excision repair	−1.88407149
KEGG pathway	hsa04657	IL-17 signaling pathway	−1.88229346
KEGG pathway	hsa05016	Huntington’s disease	−1.88122179
KEGG pathway	hsa04115	p53 signaling pathway	−1.8248744
KEGG pathway	hsa03060	Protein export	−1.82430999
KEGG pathway	hsa03018	RNA degradation	−1.73111307
KEGG pathway	hsa05031	Amphetamine addiction	−1.54416154
KEGG pathway	hsa04950	Maturity-onset diabetes of the young	−1.53379589

### Identification of Hub Genes

The scatterplot of GS (*y*-axis) vs. MM (*x*-axis) is shown in the yellow (*R* = −0.75, *p* < 1 × 10^–200^) and blue modules (*R* = 0.87, *p* < 1 × 10^–200^) (Figures 3D,E). MM had a highly significant correlation with GS in these two modules, which implies that the hub genes in the yellow and blue co-expression modules are highly correlated with infertility in endometriosis. In our study, 885 candidate hub genes (19 lncRNAs and 866 mRNAs) in the yellow module and 970 candidate hub genes (84 lncRNAs and 886 mRNAs) in the blue module were identified.

**FIGURE 4:**
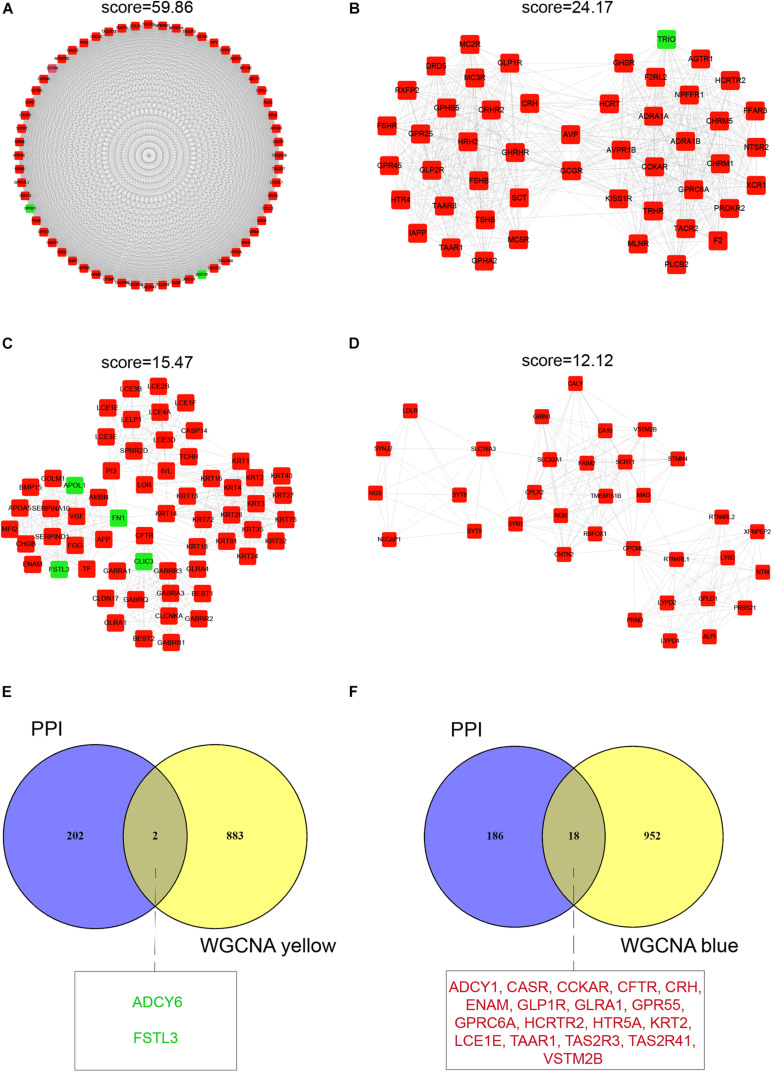
Protein–protein interaction network cluster analysis and identification of hub mRNAs. A PPI network containing 1,174 nodes and 12,150 edges was constructed by filtering the 1,990 DEGs using STRING. Shown are four clusters in the network that had the highest scores (*k*-score > 10). **(A)** Cluster 1 consists of 69 nodes and 1,766 edges. **(B)** Cluster 2 consists of 49 nodes and 652 edges. **(C)** Cluster 3 consists of 61 nodes and 464 edges. **(D)** Cluster 4 consists of 34 nodes and 200 edges. **(E,F)** Venn diagrams of common hub mRNAs in the yellow and blue modules. The nodes or hub mRNAs in green represent down-regulated genes and those in red represent up-regulated genes in infertile women with endometriosis compared with fertile women with endometriosis.

For the identification of hub mRNAs associated with infertility in women with endometriosis, we constructed a PPI network and screened four clusters comprising 204 significant candidate hub mRNAs (Supplementary Table 1) containing 60 nodes (Figure 4A), 49 nodes (Figure 4B), 61 nodes (Figure 4C), and 34 nodes (Figure 4D) using the MCODE scoring system with a threshold of *k*-score > 10. Furthermore, we compared these 204 candidate hub mRNAs in the PPI network to the hub genes screened from the modules and identified two overlapping hub mRNAs in the yellow module (Figure 4E) and 18 in the blue module (Figure 4F). These 20 hub mRNAs are listed in Table 3.

**TABLE 3:** Common hub mRNAs of WGCNA and PPI analysis in the yellow and blue modules.

	Gene description	WGCNA					PPI analysis	Limma analysis		
		Module	GS	GS *P*-value	MM	MM *P*-value	*K* Score	Log2FC	FDR	Up or Down
ADCY6	Adenylate cyclase 6	Yellow	−0.88914374	2.10176E-05	0.977029022	2.01881E-09	Cluster 1: 59.864	−4.32322549	1.62E-13	Down
FSTL3	Follistatin like 3	Yellow	−0.86138354	7.54665E-05	0.975307638	3.10296E-09	Cluster 2: 27.167	−6.06007779	1.09E-12	Down
ADCY1	Adenylate cyclase 1	Blue	0.815437991	0.000378522	0.888705991	2.14994E-05	Cluster 1: 59.864	4.540162745	8.07E-12	Up
CASR	Calcium sensing receptor	Blue	0.889887433	2.02E-05	0.851114402	0.000113203	Cluster 1: 59.865	4.154828687	0.000000007	Up
CCKAR	Cholecystokinin A receptor	Blue	0.85841125	8.51358E-05	0.892040065	1.80473E-05	Cluster 2: 27.170	5.33799687	3.77E-12	Up
CFTR	CF transmembrane conductance regulator	Blue	0.877568366	3.71619E-05	0.916502067	4.07889E-06	Cluster 3: 15.467	6.435926076	1.21E-13	Up
CRH	Corticotropin releasing hormone	Blue	0.850563948	0.000115593	0.930216776	1.43E-06	Cluster 2: 27.172	4.785431728	1.38E-11	Up
ENAM	Enamelin	Blue	0.900736051	1.11178E-05	0.941971756	4.86E-07	Cluster 3: 15.467	4.953118924	2.71E-12	Up
GLP1R	Glucagon-like peptide 1 receptor	Blue	0.850921895	0.000114034	0.866580845	6.07E-05	Cluster 2: 27.172	6.07312786	4.19E-13	Up
GLRA1	Glycine receptor alpha 1	Blue	0.839537197	0.000172792	0.878336963	3.58E-05	Cluster 3: 15.467	4.287442801	2.79E-11	Up
GPR55	G protein-coupled receptor 55	Blue	0.855998273	9.37062E-05	0.909933263	6.33E-06	Cluster 1: 59.864	4.930381688	9.49E-12	Up
GPRC6A	G protein-coupled receptor class C group 6 member A	Blue	0.832428091	0.000220505	0.918722191	3.49E-06	Cluster 2: 27.167	4.303509957	4.51E-12	Up
HCRTR2	Hypocretin receptor 2	Blue	0.836214917	0.00019392	0.915205528	4.46E-06	Cluster 2: 27.167	4.558044205	2.09E-11	Up
HTR5A	5-Hydroxytryptamine receptor 5A	Blue	0.874246266	4.33124E-05	0.952672218	1.46E-07	Cluster 1: 59.864	4.038237249	9.38E-12	Up
KRT2	Keratin 2	Blue	0.880981394	3.16052E-05	0.956618535	8.76E-08	Cluster 3: 15.467	4.448300733	4.48E-11	Up
LCE1E	Late cornified envelope 1E	Blue	0.866279363	6.1499E-05	0.957508323	7.75E-08	Cluster 3: 15.467	4.542418549	2.47E-11	Up
TAAR1	Trace amine associated receptor 1	Blue	0.839448887	0.000173328	0.947895963	2.58E-07	Cluster 2: 27.167	4.62537402	4.84E-13	Up
TAS2R3	Taste 2 receptor member 3	Blue	0.878360965	3.58053E-05	0.939606798	6.15E-07	Cluster 1: 59.864	4.106640312	5.95E-12	Up
TAS2R41	Taste 2 receptor member 41	Blue	0.905530556	8.34943E-06	0.887652146	2.27E-05	Cluster 1: 59.865	4.606795778	1.14E-11	Up
VSTM2B	V-set and transmembrane domain containing 2B	Blue	0.801793908	0.000562628	0.867303193	5.89E-05	Cluster 4: 12.121	5.226197818	4.11E-12	Up

*WGCNA, weight gene co-expression network analysis; PPI, protein–protein interaction; GS, gene significance; MM, module membership; log_2_FC, log2 (fold-change) values of differentially expressed genes between fertile and infertile women with endometriosis.*

**FIGURE 5:**
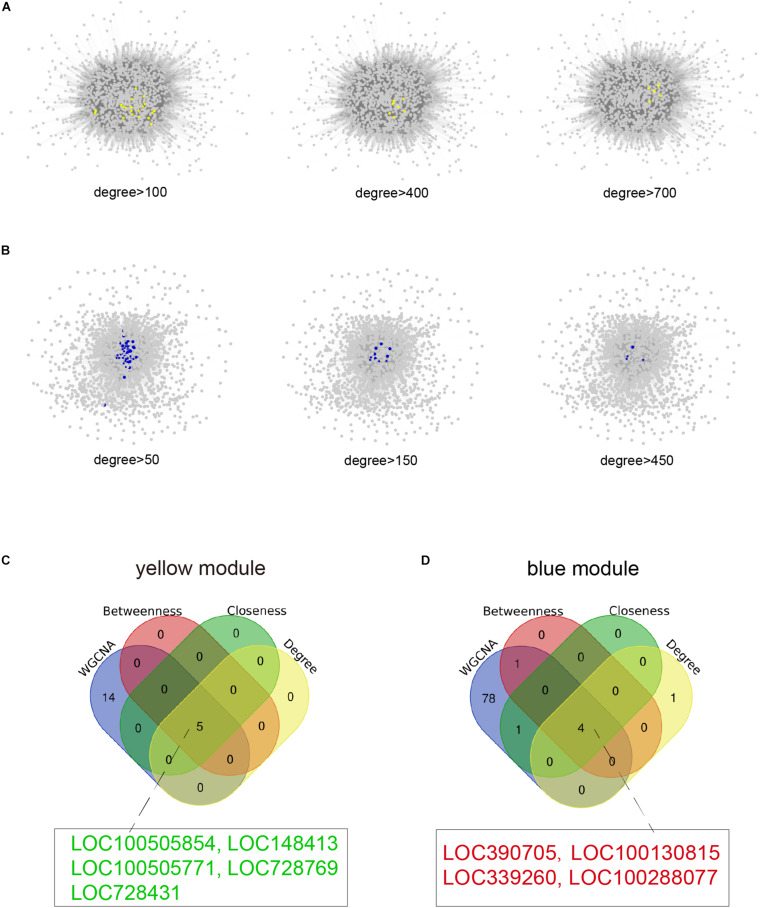
Identification of hub lncRNAs. **(A)** The lncRNAs with more than 100, 400, or 700 connections in the weighted gene co-expression network in the yellow module are highlighted as yellow dots as visualized by Cytoscape. **(B)** The lncRNAs with more than 50, 150, or 450 connections in the weighted gene co-expression network in the blue module are highlighted as blue dots as visualized by Cytoscape. **(C,D)** Venn diagrams of common hub lncRNAs among WGCNA and top five lncRNA lists of degree, betweenness, and closeness in the yellow and blue modules. The hub lncRNAs in red font represent up-regulated genes and hub lncRNAs in green font represent down-regulated genes in infertile women with endometriosis compared with fertile women with endometriosis.

**FIGURE 6:**
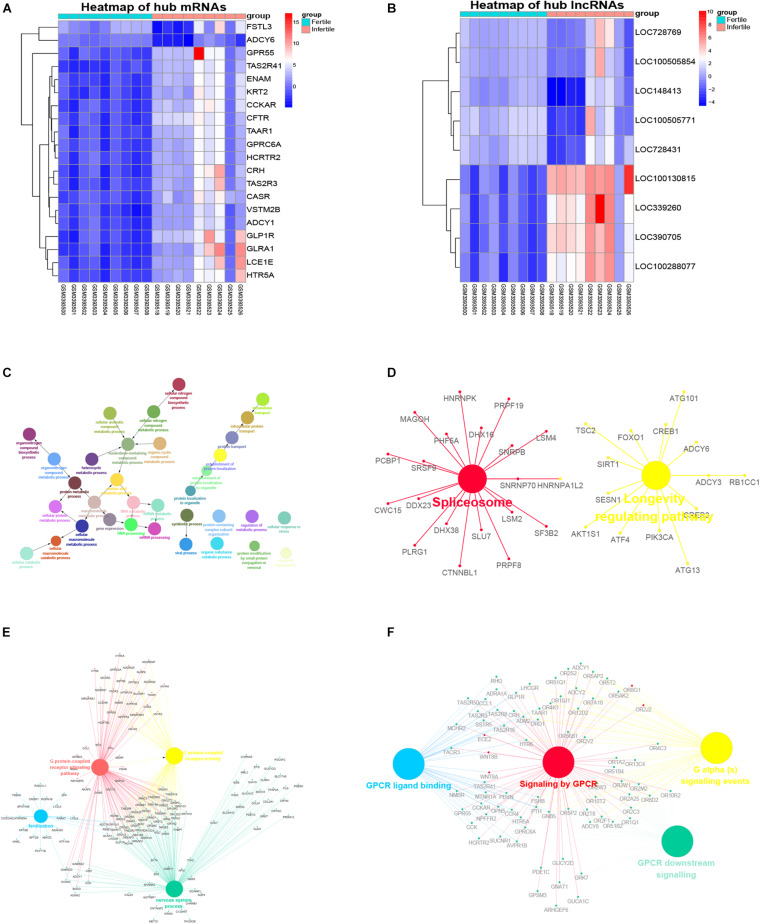
Heatmaps of the hub genes and functional predication of the hub lncRNAs. **(A)** Heatmap of the expression of hub mRNAs in infertile and fertile women with endometriosis. **(B)** Heatmap of the expression of hub lncRNAs in infertile and fertile women with endometriosis. **(C)** GO biological processes of LOC100505854 co-expressed mRNAs in the yellow module. **(D)** KEGG pathways of LOC100505854 co-expressed mRNAs in the yellow module. **(E)** GO biological processes of LOC390705 co-expressed mRNAs in the blue module. **(F)** KEGG pathways of LOC390705 co-expressed mRNAs in the blue module.

For the identification of hub lncRNAs associated with infertility in women with endometriosis, we analyzed the topological characteristics of weighted gene co-expression networks with degree, closeness, and betweenness. lncRNAs with a high degree of connectivity are closer to the center of the co-expression networks (Figures 5A,B). We identified lncRNAs with the top five degrees that also belonged to the hub genes identified by WGCNA and the top five lncRNA lists of closeness and betweenness. There were four and five lncRNAs that satisfied these criteria in the yellow and the blue modules, respectively (Figures 5C,D). The nine hub lncRNAs are listed in Table 4. Heatmaps revealed distinct expression patterns of the 20 hub mRNAs and the nine hub lncRNAs in infertile and fertile women with endometriosis (Figures 6A,B).

**TABLE 4:** Common hub lncRNAs of WGCNA and topological analysis in the yellow and blue modules.

Symbol	Module	WGCNA				Topological analysis			Limma analysis		
		GS	GS *P*-value	MM	MM *P*-value	LncRNA betweenness	LncRNA closeness	lncRNA degree	Log2FC	FDR	Up or Down
LOC100505854	Yellow	−0.8217	0.00031	0.9739	4.3E-09	8422.938722	4.25E-04	1030	−1.8039	1.48E-11	Down
LOC148413	Yellow	−0.8343	0.00021	0.96687	1.8E-08	6322.04946	4.17E-04	983	−4.5227	2.06E-13	Down
LOC100505771	Yellow	−0.9462	3.1E-07	0.98214	4.5E-10	11642.66986	3.88E-04	806	−4.1625	3.43E-14	Down
LOC728769	Yellow	−0.8604	7.9E-05	0.96763	1.5E-08	2256.936121	3.76E-04	733	−1.8701	6.79E-11	Down
LOC728431	Yellow	−0.9084	7E-06	0.97579	2.8E-09	3033.478056	3.76E-04	719	−3.5328	1.92E-12	Down
LOC100130815	Blue	0.88305	2.9E-05	0.90683	7.7E-06	12665.77631	0.000440723	459	6.85512	6.2E-14	Up
LOC100288077	Blue	0.8565	9.2E-05	0.93155	1.3E-06	972.4226098	0.000378931	99	5.05612	3.8E-12	Up
LOC339260	Blue	0.86325	7E-05	0.94982	2.1E-07	22755.95494	0.00043956	453	5.23495	1.2E-11	Up
LOC390705	Blue	0.87677	3.9E-05	0.93408	1E-06	77322.16426	0.000528262	833	6.02721	3E-13	Up

To further explore the hub genes specifically associated with EAI, the 29 identified hub genes were compared to the DEGs between the fertile and infertile women without endometriosis. The Venn diagram shows the 19 overlapping hub genes (including 14 mRNAs and five lncRNAs) that were associated with infertility irrespective of endometriosis status (Supplementary Figure 2A). Ten hub genes (including five mRNAs and five lncRNAs) were specific to EAI (Supplementary Figure 2A), suggesting that these genes might play unique roles in infertility caused by endometriosis.

### Validation of Hub Genes

Since there is no other publicly available transcriptome dataset with information about fertility status in women with endometriosis, the infertility-associated 14 overlapping hub mRNAs were selected for validation using the GSE26787 dataset in which the fertility status of the patients was known. It is unknown, however, if any of the patients had endometriosis. Eight hub genes were differentially expressed between fertile and infertile women (Supplementary Figure 3A); six genes were associated with recurrent miscarriages and two were associated with implantation failures. As expected, the specific EAI-associated five hub mRNAs were not differentially expressed in this dataset. These results support the validity of our bioinformatics analysis.

### Function Prediction of the Hub lncRNAs

The hub lncRNAs in the same module had similar potential functions (data not shown). In our topological analysis of the co-expression modules, LOC100505854 was the first-ranked lncRNA in the yellow module and LOC390705 was the first-ranked lncRNA in the blue module. Functional annotations for these two lncRNAs and co-expressed mRNAs are shown in Figures 6C–F. The GO terms and KEGG pathways of LOC100505854 were enriched in the nucleobase-containing compound metabolic process, RNA processing, and the spliceosome pathway (Figures 6C,D), while the GO terms and KEEG pathways of LOC390705 were mainly enriched in G protein-coupled receptor (GPCR) activity, fertilization, and the GPCR ligand binding pathway (Figures 6E,F).

## Discussion

Endometriosis is associated with female infertility, but the molecular mechanisms underlying infertility are poorly understood. Although current medical and surgical treatments may help treat endometriosis symptoms, such as pelvic pain and dysmenorrhea, there has been no evidence that they enhance fertility. In fact, ovulation-suppressing agents may indirectly negatively affect fertility by minimizing the window of opportunity when fertility treatments are still effective (Practice Committee of the American Society for Reproductive Medicine, 2012). Thus, it is imperative to identify diagnostic biomarkers and therapeutic targets for the effective management of infertility in women with endometriosis. Our study identified infertility-associated hub RNAs in women with endometriosis using lncRNA and mRNA expression profile data from the GEO database^4^. Our findings elucidate potential molecular alterations associated with infertility in women with endometriosis and provide a useful resource for the identification of biomarkers of infertility, which may improve specificity and accuracy in the early diagnosis and treatment of infertility in women with endometriosis.

With the improvement of omics technologies, novel qualitative and quantitative measures have been developed to evaluate various biological systems. It is estimated that biomarkers identified through the analysis of the entire underlying network structure are more robust and better reflect the involved complex biology (Sung et al., 2012). Also, integrative analysis of diverse biological networks can eliminate potential biases of single-omics analyses (Al-Anzi et al., 2017; Nangraj et al., 2020). In this study, we used the PPI network and WGCNA as multi-omics strategies to uncover infertility-related biological mechanisms. To reveal the biological basis of infertility associated with endometriosis, these networks were further evaluated based on their relationship to phenotypic traits (module analysis), MCODE score (hub analysis), or degree centrality (degree analysis). Compared to other bioinformatics methods, WGCNA is more reliable and the results have greater biological significance (Wang Y. et al., 2019). WGCNA can predict a cluster of co-expressed genes associated with a specific biological function or tissue type, and these highly correlated nodes are representative genes that contribute to a phenotype or disease. In our study, WGCNA proved to be an effective method to recognize the biologically relevant modules and diagnostic biomarkers of infertility in women with endometriosis. Further functional analysis showed that genes clustered in the yellow and blue modules were predominantly involved in infertility, which confirmed the reliability of module analysis in recognizing clinically significant genes (Landfors et al., 2016; Rumi et al., 2017; Wang W. et al., 2019). In addition, lncRNAs are emerging as promising biomarkers that can form complex networks with many genes and may regulate their co-expressed mRNAs in the modules (Liao et al., 2011). Topological parameters (degree, betweenness, and closeness) were analyzed to identify central lncRNAs in the infertility-associated co-expression networks. Additionally, the PPI network based on DEMs was constructed to identify functional gene connections. Its densely connected regions containing hub mRNAs were found by MCODE based on a scoring system. This method allowed for the identification of 20 hub mRNAs that highly correlated with infertility in the module analysis and had the highest number of connections in the PPI network.

Some of the hub mRNAs, such as TAS2R3, TAS2R41, CASR, CCKAR, GPR55, HCRTR2, CFTR, and ENAM, were also identified as gene sets with core enrichment, which confirmed the biological importance of hub mRNAs. Some hub mRNAs in this study, including CASR, CCKAR, CFTR, CRH, FSTL3, GLP1R, GPR55, and TAAR1, have been reported to play important roles in the pathogenesis of infertility. For instance, CFTR was shown to be involved in aromatase activation and estrogen production, both of which play important roles in infertility in women with endometriosis (de Ziegler et al., 2010; Chen et al., 2012). CASR could potentially influence a variety of reproductive processes, such as proliferation, maturation, or mobility of germ cells and implantation of the zygote (Ellinger, 2016). FSTL3, which was identified as a specific hub gene of EAI in our analysis, has previously been shown to be downregulated in endometriosis (May-Panloup et al., 2012) and might regulate endometrium receptivity, a key factor of EAI (Xu et al., 2020). Our findings indicate that hub mRNAs may have different roles in the mechanisms of infertility in women with endometriosis. These hub mRNAs could affect hormone levels and inflammatory status in the eutopic endometrium and cause infertility due to sperm dysfunctions and embryo implantation failure, which is consistent with the known theory of infertility in endometriosis (de Ziegler et al., 2010). We also discovered novel hub mRNAs, such as TAS2R3, VSTM2B, and HCRTR2, and other specific EAI-associated genes, such as ADCY6, ADCY1, and GLRA1, which have not been previously reported in female infertility but have been associated with important biological functions, including meiotic arrest and neuronal signaling (Mircea et al., 2007; Gentiluomo et al., 2017; Sethna et al., 2017; Dunietz et al., 2020). These identified mRNAs warrant further investigation and validation as they may elucidate novel mechanisms of infertility in endometriosis.

The diagnostic role of lncRNAs in infertility in women with endometriosis has not been studied. In our study, WGCNA was used to recognize hub lncRNAs and reveal their functions by showing lncRNA–mRNA interactions in the module. Some of these hub lncRNAs have been reported in other diseases. For instance, LOC390705 might be a candidate hub gene contributing to tumorigenesis of colorectal cancers (Chen et al., 2017). Genes in a module are closely related in function. Therefore, the lncRNAs involved in the yellow or blue modules are considered to have similar functions, which was further confirmed by our analyses. Here, we showed the potential functions of the two top-ranked lncRNAs, LOC390705 and LOC100505854. Many of these functions may be correlated with infertility in endometriosis. For example, GPCR is an important membrane protein that senses signaling molecules, such as hormones, and GPCR methylation may impair endometrial receptivity, which is an important risk factor for infertility in endometriosis (Guo, 2019; Pathare and Hinduja, 2020). Cellular response to stress, especially response to oxidative stress, including apoptosis and DNA damage, was shown to be involved in the mechanisms of infertility in endometriosis (Gupta et al., 2008). Together, these hub lncRNAs are likely to play roles in infertility in women with endometriosis by regulating functions such as GPCR activity, which was also identified in the GSEA. Our results may provide new insights into the molecular mechanisms of infertility associated with endometriosis.

Our study has several limitations. Firstly, the dataset we used for the identification of endometriosis-associated infertility genes did not have comprehensive clinical information, which may have affected the evaluation of the data. Secondly, the controversy regarding whether endometriosis, especially the milder stages of endometriosis, is a cause of infertility or merely an incidental finding is ongoing. As discussed by Gupta et al. (2008), this controversy might be due to study design limitations, including the lack of fertile endometriosis patients as controls, differences in the severity of endometriosis, and heterogeneous patient groups. To avoid or mitigate these problems, we chose the dataset consisting of stage IV ovarian endometriosis patients without any other endocrinological disorder or other complications of comorbidities. Our identified hub genes were dysregulated in infertile women with endometriosis compared to fertile women with endometriosis. Thus, the biological functions of the hub genes might be highly correlated with infertility in endometriosis. We further searched the specific EAI-associated hub genes by screening out the genes that were differentially expressed between fertile and infertile patients without endometriosis. The absolute cause-and-effect relationship as well as the unique biomarkers for EAI need to be confirmed by future experiments. Thirdly, we could not identify an independent public dataset that contained information on both fertility and endometriosis status to validate our endometriosis-specific infertility genes. However, using an independent dataset of samples collected from patients with known fertility status (unknown endometriosis status), we were able to validate infertility genes that were common to patients with and without endometriosis. It will be necessary to further confirm these identified hub genes and co-expression networks in large-scale clinical studies for application in infertility management in women with endometriosis.

Our study, for the first time, systematically identified the infertility-associated hub lncRNAs and mRNAs in women with endometriosis using the WGCNA algorithm and explored the functions of these genes. These findings provide new resources for better understanding of the pathogenesis of endometriosis-associated infertility and identification of new diagnostic/therapeutic approaches that may help predict the infertility outcome of endometriosis patients and eventually improve fertility rates.

## Data Availability Statement

Publicly available datasets were analyzed in this study. This data can be found here: https://www.ncbi.nlm.nih.gov/geo/query/acc.cgi?acc=GSE120103.

## Author Contributions

JW, XF, and SO conceived and designed the study. JW and XX analyzed the data. JW wrote the manuscript. YH and SO critically evaluated the data and contributed to the writing of the manuscript. All authors contributed to the article and approved the submitted version.

## Conflict of Interest

The authors declare that the research was conducted in the absence of any commercial or financial relationships that could be construed as a potential conflict of interest.

## Footnotes

1 http://www.bioconductor.org/packages/release/bioc/html/clusterProfiler.html

2 http://metascape.org/gp/index.html

3 http://string-db.org/

4 https://www.ncbi.nlm.nih.gov/geo/info/linking.html

## Supplementary Material

Supplementary Figure 1 Functional annotations of clinically significant modules. **(A,B)** Functional annotations including the GO biological process and KEGG pathway of genes in the yellow module. **(C,D)** Functional annotations including the GO biological process and KEGG pathway of genes in the blue module.

Supplementary Figure 2 Venn diagram of 10 specific genes for endometriosis-associated infertility. The hub mRNAs in green represent down-regulated genes and the hub mRNAs in red represent up-regulated genes in infertile women with endometriosis compared with fertile women with endometriosis.

Supplementary Figure 3 Validation of hub mRNAs in an independent dataset. Boxplots show the significantly differentially expressed hub mRNAs between fertile and infertile patients.

Supplementary Table 1 Clustering nodes in the protein–protein interaction network.
