# Supplementary figures and images for: Identification of Infertility-Associated Topologically Important Genes Using Weighted Co-expression Network Analysis
Source: Front Genet. 2021 Feb 3;12:580190. doi: 10.3389/fgene.2021.580190 (PMC7887323; doi:10.3389/fgene.2021.580190)

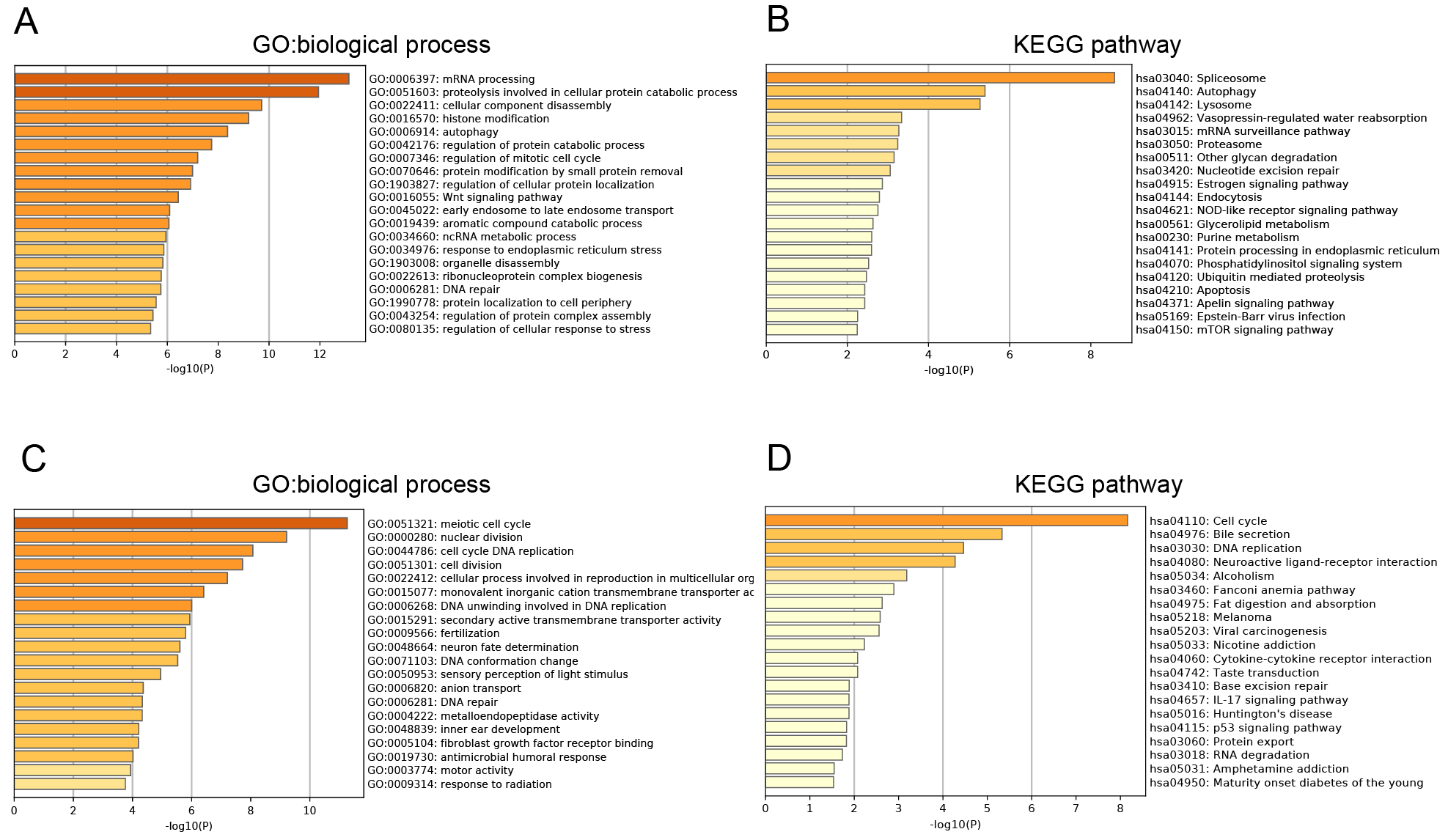

Supplement: Supplementary Figure 1 — Functional annotations of clinically significant modules. (A,B) Functional annotations including the GO biological process and KEGG pathway of genes in the yellow module. (C,D) Functional annotations including the GO biological process and KEGG pathway of genes in the blue module. [file Image_1.TIF]

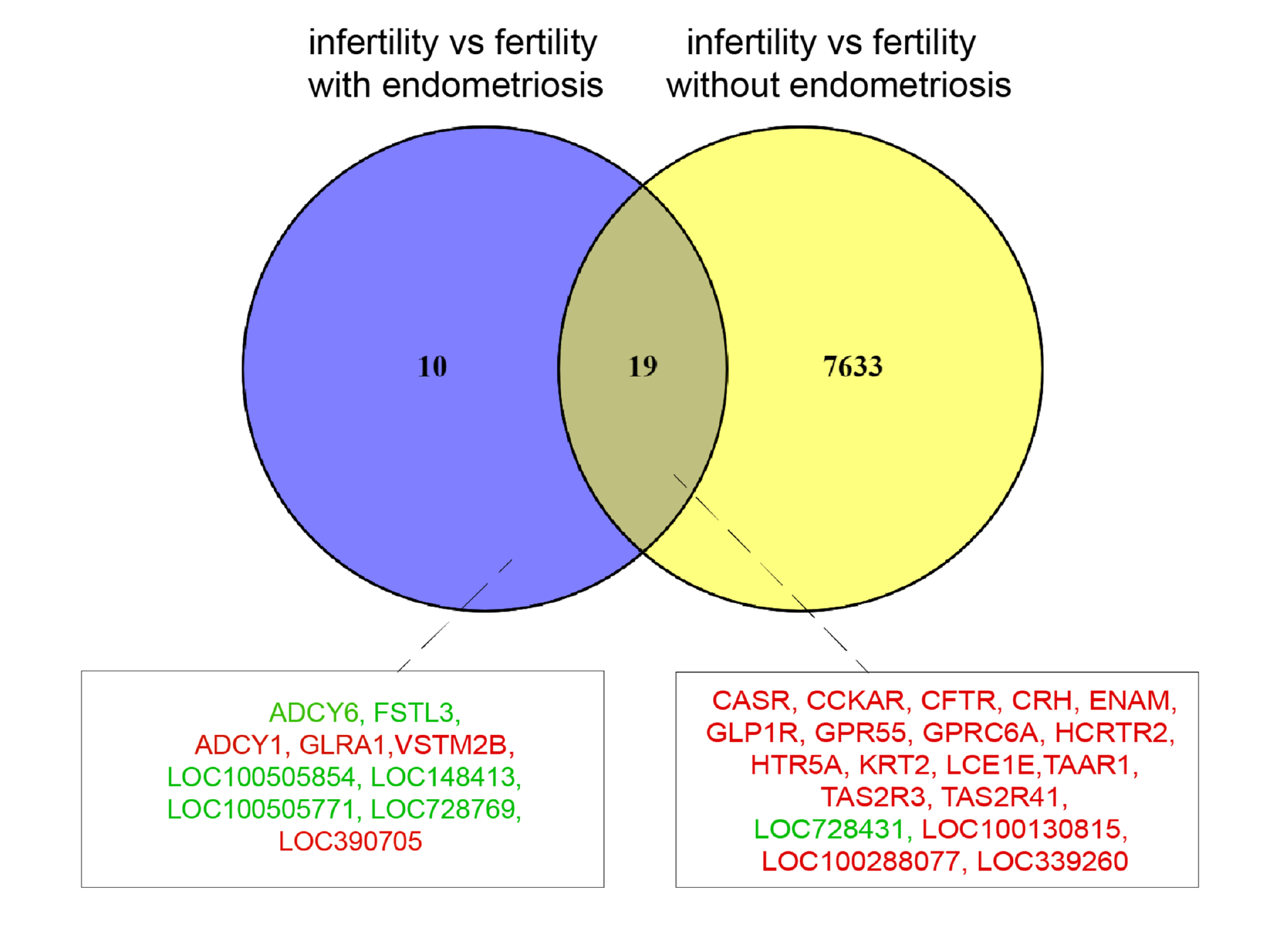

Supplement: Supplementary Figure 2 — Venn diagram of 10 specific genes for endometriosis-associated infertility. The hub mRNAs in green represent down-regulated genes and the hub mRNAs in red represent up-regulated genes in infertile women with endometriosis compared with fertile women with endometriosis. [file Image_2.TIF]

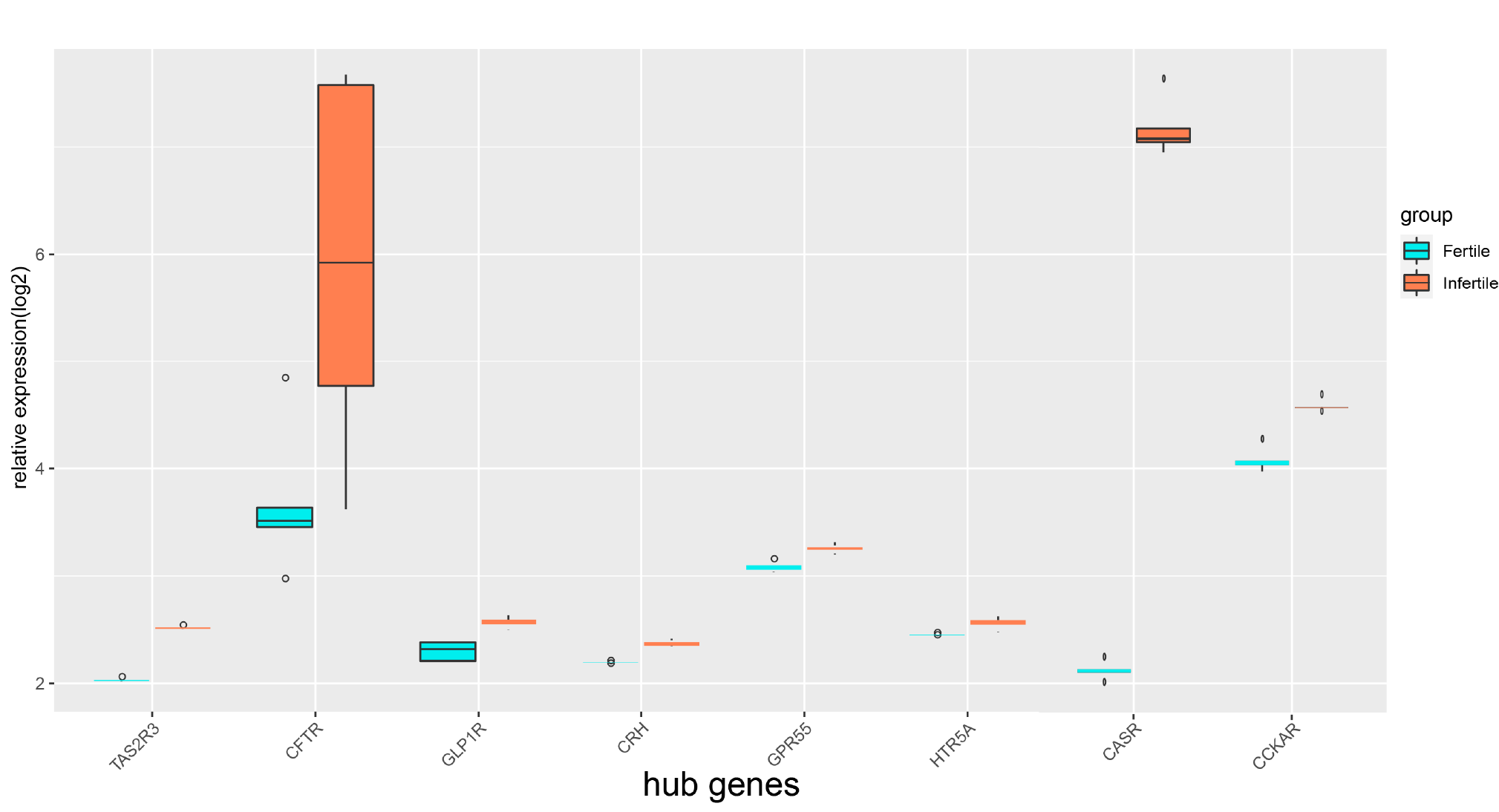

Supplement: Supplementary Figure 3 — Validation of hub mRNAs in an independent dataset. Boxplots show the significantly differentially expressed hub mRNAs between fertile and infertile patients. [file Image_3.TIF]
